# Construction of Hybrid Dual Radio Frequency RSSI (HDRF-RSSI) Fingerprint Database and Indoor Location Method

**DOI:** 10.3390/s20102981

**Published:** 2020-05-24

**Authors:** Haotai Sun, Xiaodong Zhu, Yuanning Liu, Wentao Liu

**Affiliations:** College of Computer Science and Technology, Jilin University, Changchun 130012, China; s88500860@163.com (H.S.); zhuxd@jlu.edu.cn (X.Z.); amor_lwt@163.com (W.L.)

**Keywords:** deep learning, fingerprint location, hybrid dual radio frequency fingerprint, indoor and outdoor location, location based services (LBS), physical distance, RSSI, signal distance

## Abstract

Radio frequency communication technology has not only greatly improved public network service, but also developed a new technological route for indoor navigation service. However, there is a gap between the precision and accuracy of indoor navigation services provided by indoor navigation service and the expectation of the public. This study proposed a method for constructing a hybrid dual frequency received signal strength indicator (HDRF-RSSI) fingerprint library, which is different from the traditional RSSI fingerprint library constructing method in indoor space using 2.4G radio frequency (RF) under the same Wi-Fi infrastructure condition. The proposed method combined 2.4G RF and 5G RF on the same access point (AP) device to construct a HDRF-RSSI fingerprint library, thereby doubling the fingerprint dimension of each reference point (RP). Experimental results show that the feature discriminability of HDRF-RSSI fingerprinting is 18.1% higher than 2.4G RF RSSI fingerprinting. Moreover, the hybrid radio frequency fingerprinting model, training loss function, and location evaluation algorithm based on the machine learning method were designed, so as to avoid limitation that transmission point (TP) and AP must be visible in the positioning method. In order to verify the effect of the proposed HDRF-RSSI fingerprint library construction method and the location evaluation algorithm, dual RF RSSI fingerprint data was collected to construct a fingerprint library in the experimental scene, which was trained using the proposed method. Several comparative experiments were designed to compare the positioning performance indicators such as precision and accuracy. Experimental results demonstrate that compared with the existing machine learning method based on Wi-Fi 2.4G RF RSSI fingerprint, the machine learning method combining Wi-Fi 5G RF RSSI vector and the original 2.4G RF RSSI vector can effectively improve the precision and accuracy of indoor positioning of the smart phone.

## 1. Introduction

At present, wireless networks and mobile smart terminals play crucial roles in human life. With the help of various smart terminals and derivative application services, humans have gradually entered a new era of mobile Internet information. In particular, the deep integration of mobile smart terminals and location-based services has greatly affected and changed traditional human lifestyles in all aspects. As a key node that connects the online and offline worlds, location based services (LBSs) play a key role in practical applications.

The study of location-based service technology started in the 1990s [1]. At present, LBS, as one of the most important basic supporting technologies in the information industry, has been widely applied in the fields of human life service [2,3], industry [4], economy [5], social networking [6], and emergency rescue [7]. In addition, it has broad commercial prospects. According to statistics, the global market of LBS will grow at a compound annual growth rate of 42% in the next few years. The market size is expected to expand from US $7 billion 110 million in 2017 to US $40 billion 990 million in 2022 [8].

In outdoor space, the construction and industrial application of the four major satellite positioning systems has been realized through unremitting efforts of various countries. The positioning accuracy of the global navigation satellite system can meet the location-based service demand in the military and civil fields [9]. Due to the shelter of building walls, global navigation satellite system (GNSS) signals cannot penetrate walls into the indoor space, which limits the application of GNSS in indoor space positioning. Meanwhile, the positioning range is relatively small in indoor space, and positioning accuracy is an important evaluation index for indoor positioning application. For example, positioning targets may occur in different rooms because of the errors of 2–3 m. Wrong positioning results indicate the failure of positioning. Therefore, there are higher requirements for the accuracy of indoor positioning than outdoor positioning. According to a report from Nokia, most people spend more than 80% of their time in indoor spaces [10]. In recent years, the number of high-rise buildings and large-scale urban complexes has increased. Therefore, developing LBSs for indoor spaces is urgently needed. As location technology is the basis for location services, it is still challenging to rapidly and accurately obtain indoor location target positions. Additionally, the accuracy of the indoor location is the key technical problem that hinders industrial applications. The theory, technology, and application of indoor space location have become hot research topics for the academic world, industry, and public safety.

Scholars have carried out various studies of indoor positioning technology. The theoretical study and product development of mainstream positioning technology based on infrared ray [11], ultrasonic wave [12], inertial navigation [13], radio frequency identification (RFID) [14], Bluetooth (Bluetooth) [15], Wi-Fi [16,17], ultra wide band (UWB) [18], and image recognition [19] have made significant progress [20]. Since Wi-Fi wireless networks are widely deployed in civil and commercial buildings, additional infrastructure is not needed to provide indoor positioning service. The implementation cost of wireless positioning technology, particularly indoor location technology based on Wi-Fi wireless signals, is lower compared with other positioning technologies. Therefore, the theoretical study results and product application have a large market share. However, indoor location technology based on Wi-Fi radio frequency signal has its theoretical deficiencies. First, the spectrum 2.4 and 5 GHz of the Wi-Fi network belong to high frequency spectrum. High frequency radio signals are likely to be disturbed by external environment, especially the 2.4 GHz radio frequency signals. Human bodies have obvious absorption and attenuation effects on 2.4 GHz signals. Second, there are different kinds of building materials and complex structures in modern buildings, which obstruct the propagation of Wi-Fi signals in buildings. The reflection, refraction, and shadow fading effect of Wi-Fi signals in indoor transmission hinder the improvement of indoor location accuracy of Wi-Fi radio frequency signals. Indoor location accuracy of Wi-Fi radio frequency signals is usually 2–3 m, and the positioning accuracy is within 60% [15,16].

Based on the high applicability of the fingerprint library matching method, this paper designs the framework of a fingerprint positioning method based on hybrid dual frequency received signal strength indicator (HDRF-RSSI) according to the basic principles of the fingerprint library matching method. The dual radio (2.4G and 5G) broadcast characteristics of Wi-Fi AP networking are used to collect the dual radio RSSI characteristics of the reference node during the offline phase to fuse the dual radio characteristics. Compared with the single RF fingerprint database construction method, the hybrid dual RF RSSI fingerprint library construction method designed in this paper enhances the fingerprint density. In addition, a neural network processing method is introduced in this paper. The dual radio frequency RSSI fingerprint is used as the neural network input. The output is defined as the distance of the node to be located from the origin of the scene. The impact of non-visual conditions on the positioning is assessed.

## 2. Related Work

In 1994, American scholar Schilit first put forward three major goals of location service: where you are (spatial information), who you are with (social information), and nearby resources (information inquiry) [21]. Considering the three major goals of location service, scholars have carried out various studies of indoor positioning technology and proposed different positioning methods. With the advantages of low cost, high precision, and wide spread, Wi-Fi indoor location technology has become one of the mainstream indoor location technologies. In 2000, Microsoft Corp established the location system based on radio frequency signal fingerprint matching radio-frequency (RF)-based system [22]. The system collects RF RSSI at the specified location, uses RSSI mean to establish fingerprint database, and realizes positioning by means of K nearest neighbor (KNN). The positioning accuracy is 2–3 m. Ji et al. [23] proposed a novel and automated location determination method, which integrates ray tracing technology but ignores the influence of diffraction and scattering. Because of channel attenuation, multipath interference, and pedestrian interference, it is difficult to construct a refined path loss model in the indoor environment, and the positioning accuracy is 4 m. Roos et al. [24] used Gauss kernel function to estimate the probability density of the RSSI signal, obtained the location results using the Bayes-based method. The positioning accuracy is 3 m. Rizos et al. [25] studied the influence of K value on indoor positioning accuracy in the KNN positioning method, finding that the location effect was better when the K was 5. However, the indoor environment is complex and changeable, and a fixed K value may reduce the positioning accuracy, leading to low universality. Shin et al. [26] proposed a method to select reference points using Euclidean distance, improved the weighted K nearest neighbor (WKNN) localization method, and realized the dynamic selection of K value. The positioning accuracy is 2.5 m. Wang et al. [27] used the deep neural network in the indoor location, studied the fingerprint-based indoor location method under the Wi-Fi network, and established a deep learning model. Based on the depth learning algorithm, they established an indoor location fingerprint matching library using the estimated angle of arrival and mean amplitude dual modal data. The location error in the indoor environment is 2.2 m.

According to the existing literature, fingerprint localization based on RSSI has achieved abundant results [28,29], but there are few results of fingerprint localization based on geomagnetic vector and radio frequency Channel State Information (CSI) vector [30]. The main reason is that the geomagnetic vector characteristics are seriously affected by the surrounding environment, and the jump is not uniform in time domain, leading to low accuracy. The geomagnetic fingerprint library and other location technologies are generally combined in order to achieve a higher positioning accuracy. Construction of the fingerprint library using the RF CSI vector has great problems in universality. Most Wi-Fi RF chips or modules do not provide access to CSI information, making is difficult to achieve universality in practicality. Applying the matrix antenna to obtain RF transmitting and receiving angles increases the complexity of deployment and the difficulty of popularization. Considering the universality of the Wi-Fi hotspot application, it is the most economical and feasible way to use Wi-Fi RF RSSI to establish the fingerprint library. However, there are still many problems in RSSI mode, which are dependent on the transmission characteristics of radio frequency. The reflection, refraction, interference, and shadow fading of radio frequency make the RSSI value of a certain location inconsistent. There are also different characteristics in time domain. Many studies focus on how to collect RF RSSI, load a variety of filters, eliminate the jump of signals, and simultaneously process data weighting. Using temporal and spatial constraints, data are processed, expecting the fingerprint library can characterize the uniqueness of each bit.

**Figure 1:**
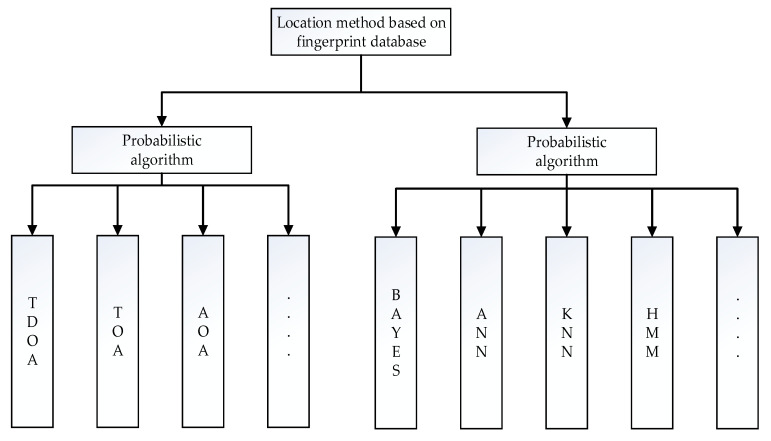
Deterministic positioning method and probabilistic positioning method.

At present, the representative classification method divides location estimation algorithms based on fingerprint library positioning technology into deterministic algorithms [22,26] and probabilistic algorithms [24,25,27], as shown in Figure 1. Deterministic algorithms include Time of arrival (TOA) [31], Time difference of arrival (TDOA) [32], Angle of arrival (AOA) [33]. The theoretical basis of deterministic algorithms is described as follows: according to different propagation velocities of electromagnetic waves in different media and the RSSI difference between the test point and the reference point, the distance between the two points is determined by the electromagnetic wave propagation model, so that the distance between multiple reference points and the test point can be obtained. Finally, the coordinates of the test point are calculated based on certain principles. Due to the multipath effect of radio waves propagating in specific indoor spaces, it is difficult to use a scientific radio wave propagation model to convert the signal distance between the test point and the reference point into a physical distance. Moreover, differences of various indoor structures also result in the poor universality of deterministic algorithms. Probabilistic algorithms include Bayes algorithm [34], maximum likelihood estimation [35], KNN [36]. Probabilistic algorithms analyze the probability distributions of the observation points and the reference points. First, a metric for the distance between fingerprints is defined to calculate the distance between the observed signal vector and all fingerprint records in the fingerprint database. The smaller the distance, the higher the similarity, and vice versa [37]. Finally, one or several fingerprint records with the highest matching degrees (the smallest distances) is selected, and the weighted average of the position coordinates corresponding to one or several fingerprints is regarded as the estimation result.

In the probabilistic algorithm research with the gradual maturity of artificial intelligence technology, machine learning and deep learning technology have made great progress in indoor positioning. In deep learning [38] and machine learning [39,40], the mapping relationship between the fingerprints and the position of the RSSI signal is established through kernel function. Low-dimensional nonlinear data is mapped to high-dimensional space, so as to find the linear relationship between variables. Therefore, the algorithms generally have high positioning accuracy. Some scholars [38] use neural network to identify people’s walking posture in the indoor environment, and obtain higher accuracy. There are also some scholars [39] who use neural network to fit and classify the RP node data, and apply the model to position estimation after fitting training, which has achieved excellent results. In addition, some institutions [40] schematize the RSSI radio frequency vector, use the mature neural network model to identify and classify radio frequency vector diagrams, and predict the location of the indoor target. The application of artificial intelligence, machine learning, and deep learning provides better methods for extracting fingerprint characteristics. The existing typical position estimation algorithms mainly include k nearest neighbors (KNN) [41], weighted K-nearest neighbor [37], artificial neural networks (ANN) [42], hidden Markov model (HMM) [43].

The above analysis reveals that to improve the accuracy and precision of indoor locations, the location technology or methods based on the fingerprint database focus on the following three aspects. First, how to make the reference point fingerprint database established in the offline phase cover the signal change characteristics of the reference point to enhance the fingerprint density and uniqueness. Second, how to effectively express the RSSI vector. Since RSSI only has space for semaphore changes and changes with time, the fingerprint vector established just using the RSSI value cannot cover its changes in the time domain, leading to deviations in location estimation. Third, how to estimate node location when the location node and RF transmitting node are invisible. The basic principle of fingerprint positioning is to reflect the attenuation response of a signal in radio frequency propagation in a spatial medium according to the change in the RSSI value over a spatial distance and to estimate the change in distance based on the inverse signal difference. However, the radio frequency should be propagated in the same medium, which is a prerequisite. If the radio frequency propagates in multiple media or the multipath effect is considered, there will be large errors in the signal difference, greatly decreasing the accuracy of the position estimation.

The remaining sections of this article are as follows. In Section 2, we introduce the current state of the technology development, research hotspots, and positioning algorithms for the fingerprint library matching and positioning method. In Section 3, we introduce in detail the method fusing 2.4G with 5G to form a hybrid dual RF RSSI fingerprint. The fingerprint characteristics of the database are analyzed. The 2.4G and 5G channel interference are analyzed, and the distinguishability between the reference point 2.4G-RSSI fingerprint and the dual-radio frequency RSSI fingerprint characteristics is compared. In Section 4, we introduce the framework structure of the dual radio frequency RSSI fingerprint positioning method. In detail, the RSSI fingerprint processing method, the dual radio frequency RSSI fingerprint database construction method, the construction and training methods of the dual radio frequency RSSI fingerprint machine learning model, and the location evaluation algorithm are discussed in detail. In Section 5, we design experimental scenarios for a hybrid dual radio frequency based method. The RSSI fingerprint positioning method is tested, and the test results are compared and analyzed. Finally, in Section 6, we summarize the conclusions of this article.

## 3. Analysis of 2.4G and 5G Interference Hybrid Rf Fingerprint Characteristics

Herein, the framework of an hybrid dual frequency received (HDRF) RSSI fingerprint location method is rationally designed, and a 2.4G and 5G dual radio frequency RSSI fingerprint database is constructed based on the broadcast characteristics of the Wi-Fi dual radio frequency (2.4G and 5G). To deeply understand the fingerprint characteristics of the 2.4G and 5G HDRF RSSI fingerprint database, the noise distribution of the Wi-Fi 2.4G and 5G RSSI and the distinguishability of RSSI vector features at adjacent locations are compared in this section. According to the experimental results, the RSSI fingerprint database constructed with the 2.4G and 5G dual radio frequency improves the density and distinguishability of fingerprints compared with that of the 2.4G radio frequency, which can provide more accurate data support for the subsequent location evaluation.

### 3.1. Noise Distribution of 2.4G and 5G Signals

**Figure 2:**
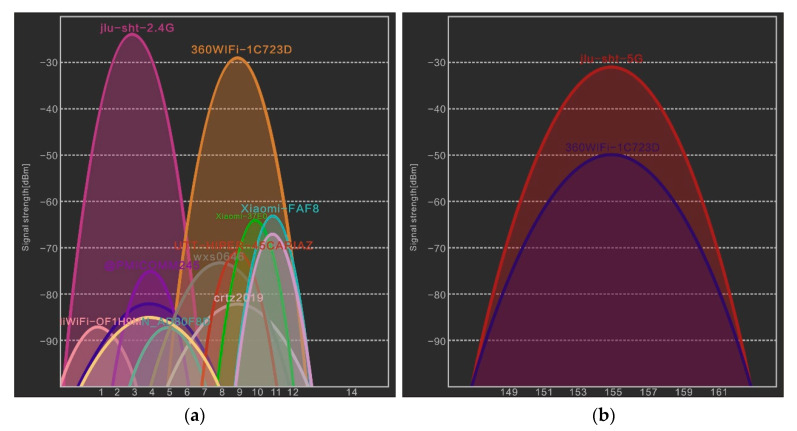
(**a**) Noise interference of Wi-Fi (2.4G) channel; (**b**) Noise interference of Wi-Fi (5G) channel noise interference.

Because the 2.4G frequency band has universal applications and is a license-free frequency, there is a lack of uniformity in indoor coverage, leading to more interference in the 2.4G band frequency [44], as shown in Figure 2a. Mutual interference may be induced by the lack of channel planning between Wi-Fi AP nodes and the simultaneous application of Wi-Fi, Bluetooth, and ZigBee, which result in errors in the signal strength received by mobile terminals. Therefore, the signals are extremely unstable, and the location results are inaccurate, which decrease the accuracy.

To address the problems of few frequency points and mutual interference in the 2.4G frequency band, 5 GHz wireless communication technology has been employed. The 5 GHz frequency band is an open ISM frequency band with a higher frequency than 2.4 GHz. Recently, this technology entered the product development stage. Moreover, it complies with international standards such as IEEE 802.11a, FCC Part 15, ETSI EN 301 489, ETSI EN 301 893, EN 50385, and EN 60950. IEEE 802.11a works in the 5G band with a frequency range of 5.725G–550 GHz, a total of 125 M of bandwidth, and 20 MHz per channel. Figure 2b shows that the 5G band is cleaner compared with the 2.4G band, leading to lower co-channel interference.

### 3.2. Distinguishability of Rssi Vectors at Adjacent Positions

According to the free space signal loss model, different RSSI should be derived at two different locations for the same AP point, and the change in RSSI observations is proportional to the logarithm of the distance in an ideal situation [45]. However, due to the complexity of indoor environments and the impact of pedestrians, non-unique RSSI values at a certain point are induced from the reflection, refraction, interference, and shadow fading of the wireless radio frequency. That is to say, the RSSI values randomly fluctuate within a certain range. The RSSI value of a single AP point cannot effectively distinguish the locations of observation devices. Therefore, the RSSI values from multiple AP points are usually integrated into a multi-dimensional vector by the fingerprint location method, which is taken as the characteristic data to distinguish a specific position from other positions like a unique fingerprint. That is why they are called RSSI fingerprint data. In the RSSI fingerprint location method, the accuracy and precision of the location estimation are closely related to the distinguishability of the fingerprint data (RSSI vectors) at adjacent locations. In general, when the distinguishability of the RSSI vectors at any two adjacent positions is stronger, the probability that the algorithm makes a wrong position estimation is smaller, and the positioning accuracy is higher.

To verify the distinguishability of 2.4G and 5G HDRF RSSI fingerprints for adjacent location fingerprints compared to traditional 2.4G radio frequency RSSI fingerprints, two experiments with the same sampling scenario, number of APs, collection equipment, and RP positions are designed in this study.

**Figure 3:**
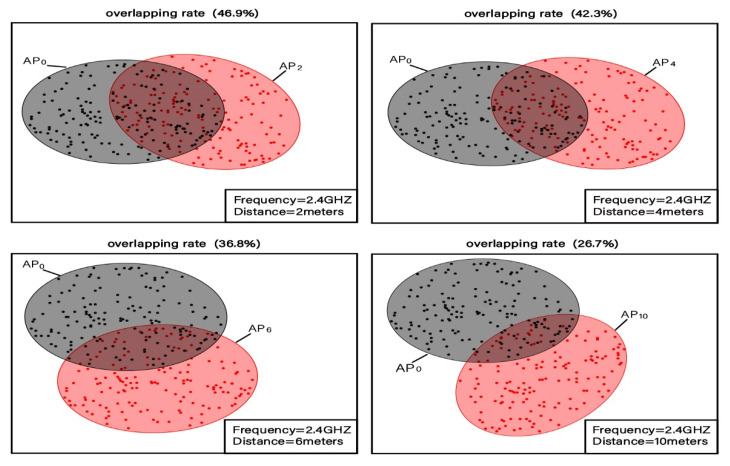
Distinguishability of 2.4G 2D fingerprint data clustering intervals of reference points at adjacent locations.

In the first experiment, there are 2 APs and 4 pairs of adjacent distance reference points. In addition, the distance between each pair of reference points is 2, 4, 6, and 10 m, respectively. The fingerprint for each reference point consists of 2D 2.4G-RSSI. A Huawei Mate 30 mobile phone is used for the 2.4G-RSSI fingerprint collection, and 150 sets of fingerprints are collected for each reference point. Figure 3 reveals the clustering distribution of the 2D fingerprints for 4 pairs of adjacent distance reference points. It can be seen that the distance between each pair of reference points is 2, 4, 6, and 10 m, respectively. As shown in Figure 3, the clustering intervals of the adjacent reference points at different distances have distinct degrees of overlap. The overlapping rates of fingerprints with a distance of 2, 4, 6, and 10 m are 46.9%, 42.3%, 36.8%, and 26.7%, respectively, and the average RSSI fingerprint clustering overlap rate is 38.1%.

**Figure 4:**
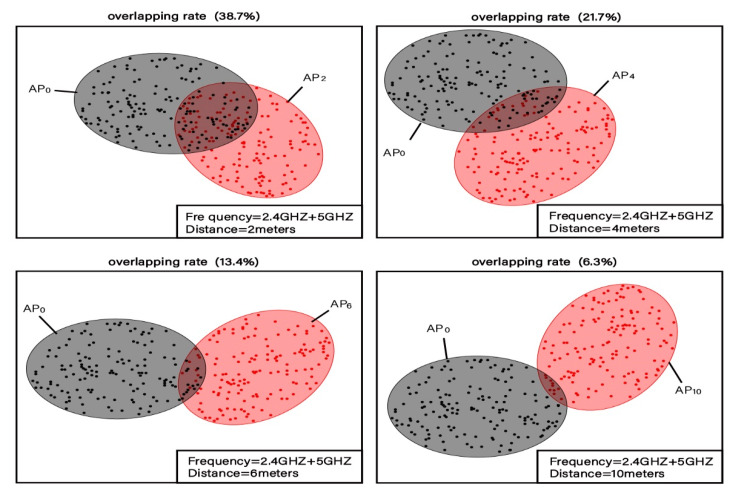
Distinguishability of 2.4G and 5G four-dimensional fingerprint data clustering intervals of reference points at adjacent positions.

In the second experiment, the original 2.4G-RSSI vector was fused with the 5G-RSSI vector of the same AP. In this way, the reference point fingerprint was constructed using four-dimensional fingerprint data. Similarly, a 150 fingerprint set was collected for each reference point. Figure 4 shows the clustering distribution of the fingerprint data for the four pairs of reference points displayed. In Figure 3, the overlapping rates of the clustering intervals of adjacent reference points with different distances are 38.7%, 21.7%, 13.4%, and 6.3%, respectively. The overlapping rate of the average RSSI vector intervals is 20.1%.

### 3.3. Advantages of Integrating 2.4G and 5G to Establish Hybrid Dual Radio RSSI Fingerprint

Based on the above two experiments, we find that 2.4G and 5G HDRF RSSI fingerprints not only enhance the density of fingerprint vectors but also decrease the overlap rate of clustering intervals of fingerprint data at adjacent locations under the same number of APs. The overlap rate is decreased by 18.1% on average, with the highest decrease being 20.4%. The stronger the distinguishability of the RSSI vectors at any two adjacent positions is, the less likely the position estimation algorithm to make an incorrect estimation, meaning higher location accuracy and precision.

## 4. The Framework of the HDRF RSSI Fingerprint Location Method

**Figure 5:**
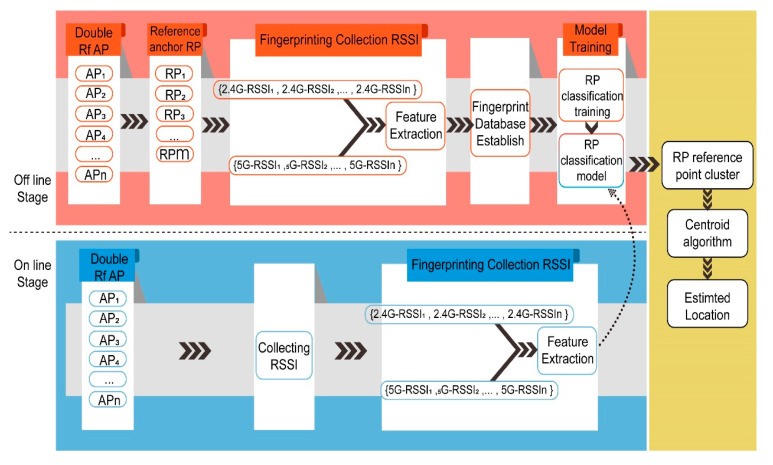
The schematic diagram of the hybrid dual frequency received signal strength indicator (HDRF-RSSI) fingerprint location method.

There are two stages in the HDRF RSSI fingerprint location process, including the offline stage and online stage. In the offline phase, it is necessary to set a number of reference positioning points (RPs) first and then use a smartphone to collect the dual radio frequency RSSI. Then, the collected RSSI signals are subject to pre-processing, and a dual-frequency RSSI fingerprint model is established. Machine learning methods are used to train the fused RSSI fingerprints. Finally, a fingerprint prediction model is generated. In the online stage, the RSSI fingerprints at the location test points (TPs) are collected and input into the fingerprint prediction model. The physical distance between the test point and the original point is set as the output. The schematic diagram is shown in Figure 5.

### 4.1. Rssi Signal Preprocessing

At present, 2.4G and 5G networks can support almost all smartphones. Therefore, mobile APP software is employed to obtain the WI-FI-AP information around the reference point. The RF RSSI data of the reference point has three parts, including the 2.4G and 5G RF signal values and data labels. 2.4G and 5G RF signals are the input of the subsequent hybrid RSSI fingerprint training model, and the data label is the distance from the reference point to the original point, which is described as follows:

(1) $$RP_{RSSI}=\left\{2.4G_{RSSI},\;5G_{RSSI},\;\mathrm{Distance}\right\}$$

$2.4G_{RSSI}=\left\{2.4G-{\mathrm{RSSI}}_{1},\;2.4G-{\mathrm{RSSI}}_{2},\;2.4G-{\mathrm{RSSI}}_{3},\;\ldots \ldots 2.4G-{\mathrm{RSSI}}_{n}\right\}$, where n is the number of dual radio frequency APs.

$5G_{RSSI}=\left\{5G-{\mathrm{RSSI}}_{1},\;5G-{\mathrm{RSSI}}_{2},\;5G-{\mathrm{RSSI}}_{3},\;\ldots \ldots 5G-{\mathrm{RSSI}}_{n}\right\}$, where, similarly, n is the number of dual radio frequency APs.

Assume the coordinates of $RP_{i}$${\mathrm{RP}}_{i}$ are $RP_{i}\left(x_{i},y_{i}\right)$, the origin of the coordinate system is (0,0), and $RP_{i}$ Distance $=\sqrt{x_{i}^{2}+y_{i}^{2}}$.

The received signal strength indicator (RSSI) is defined as the received AP signal strength indicator, which is mainly used to evaluate the link quality. The RSSI value is an interval indicator relative to the highest and lowest values. During the data collection process, the sampling frequency is set to 1 Hz, the period of each reference point is 5 min, and 300 samples in total are collected. According to Formula (2), the original data are merged to reflect the characteristics within the sampling period. Afterward, the collected RSSI data are regularized to retain the valid data and eliminate the invalid data. The data are processed based on Equation (3), and each RSSI value is 0 or 1:

(2) $$RSSI_{\mathrm{orig}}=\frac{1}{300}\sum_{i=1}^{300}RSSI_{i}$$

(3) $$RSSI_{Des}\left\{ \begin{array}{l} 1,\frac{RSSI_{orig}-RSSI_{\min}}{RSSI_{\max}-RSSI_{\min}}>\frac{1}{2}\\ 0,\frac{RSSI_{orig}-RSSI_{\min}}{RSSI_{\max}-RSSI_{\min}}\leq \frac{1}{2} \end{array}\right.$$

(4) $$RP_{i}=[\begin{array}{cccc} 2.4G-RSSI_{\left(1,1\right)} & & 2.4G-RSSI_{\left(1,n\right)} & 5G-RSSI_{\left(1,1\right)}\\ 2.4G-RSSI_{\left(2,1\right)} & \ldots \ldots & 2.4G-RSSI_{\left(2,n\right)} & 5G-RSSI_{\left(2,1\right)}\\ 2.4G-RSSI_{\left(3,1\right)} & & 2.4G-RSSI_{\left(3,n\right)} & 5G-RSSI_{\left(3,1\right)}\\ & & & \vdots \\ 2.4G-RSSI_{\left(j,1\right)} & \ldots \ldots & 2.4G-RSSI_{\left(j,n\right)} & 5G-RSSI_{\left(j,1\right)} \end{array}\begin{array}{cccc} & & & 5G-RSSI_{\left(1,n\right)}\\ & \ldots \ldots & & 5G-RSSI_{\left(2,n\right)}\\ & & & 5G-RSSI_{\left(3,n\right)}\\ & & & \vdots \\ & \ldots \ldots & & 5G-RSSI_{\left(j,n\right)} \end{array}]$$

where *i* refers to the *i*-th reference point, *j* is the *j*-th characteristic fingerprint datum at the *i*-th reference point, and n represents the number of dual radio frequency APs. Thus, hybrid RF multi-feature data over multiple time periods at the reference point can be obtained.

### 4.2. Construction of the Hybrid Rssi Fingerprint Model 

The hybrid RSSI fingerprint classification model is the CIFAR-10 model framework based on the image classification [46], which contains 3 volume layers, 3 pooling layers, 2 fully connected layers, and 1 Softmax layer. In this study, each dual RF RSSI data sample on RP is transformed to 32 × 32 image data as the input to the training model. The classification output of the CIFAR-10 model is used as the probability ratio of the distance from the observation point to the origin.

**Figure 6:**
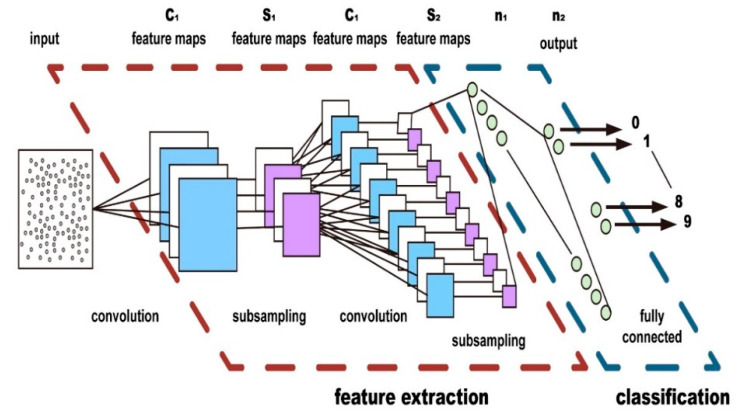
CNN structure diagram of model.

The commonly used network structure of the Cifar-10 model consists of several volumes, pooling layers, and fully connected layers, as shown in Figure 6 The volume layers are the most critical part of Convolutional Neural Networks (CNN), which mainly extract features. The pooling layers decrease the resolution of the image, that is, dimension reduction, thereby reducing the parameters of network training. The fully connected layer is usually at the end of the network and used for classification.

In this study, each dual RF RSSI data sample for RP was transformed into a 32 * 32 image as input to the training model. In the model output, the classification output of the CIFAR-10 MODEL 10 was used to calculate the probability ratio of the distance from the observation point to the origin. The CIFAR-10 model was employed to predict the probability distribution of the distance from the unknown node to the coordinate origin. The empirical probability quality function P stands for the given true distance distribution:

(5) $$P=\left[p_{d1},p_{d2},\ldots .,p_{dN}\right]\;(d1\leq di\leq dN,N=10)$$

where di is the ith distance distribution category and N refers to the number of distance distribution categories. Since $\sum_{i=1}^{N}p_{di}=1$, $p_{di}$ refers to the probability that the distance distribution falls in the *i*-th distance distribution classification. Hence, under a given probability quality function P, the average distribution score of the distance distribution can be expressed as follows:

(6) $$\mu =\frac{1}{N}\sum_{i=1}^{N}p_{di}\times di$$

The standard deviation score for the distance distribution is expressed as follows:

(7) $$\sigma =(\sum_{i=1}^{N}(di-\mu {)}^{2}\times p_{di}{)}^{1/2}$$

In Section 4.1, the dual RF RSSI fingerprint data for each reference point contain multiple 32 ∗ 32 “image data” and one distance datum. It is assumed that the farthest distance from the original point of the coordinate is $L_{max}$ and that $L_{i}$ is the distance from the reference point i to the original point. The distance can be converted to $P_{i}$ (distance distribution rate), i.e., $P_{i}=\frac{L_{i}}{L_{max}}$. The probability mass function $\hat{P}$ is expected to be found and is used to accurately estimate P. The loss function used in the fingerprint positioning model training is introduced in the following sections.

### 4.3. Loss Function

Among the various machine learning classification tasks, the Softmax cross-entropy is widely used as the training loss. $\sum_{i=1}^{N}-p_{di}\log({\hat{P}}_{di})$ refers to a loss quantization function and indicates the maximized prediction probability of correct labels, where ${\hat{P}}_{di}$ indicates the probability of falling in the ith classification. However, the cross-entropy loss cannot reflect the “distance” relationship between classifications in an ordered classification task. Shao et al. [38] suggested that (earth mover distance) EMD-based loss functions can be applied during the data training for intrinsically ordered data sets between classifications. The EMD-based loss function calculates a misclassification penalty based on the inter-class distance, which can improve the model training performance.

In this study, the distance from the TP to the set original point is predicted. The maximum distance $L_{max}$ is equally divided into ten parts to form an ordered distance distribution, namely, d1≤…≤dN(N=10). The inter-class distance r-norm can be expressed as dj−dir(1≤i,j≤10). Assume that in N ordered classification tasks with the inter-class distance of dj−dir, the quality functions of the true classifications and predicted classifications are P and $\hat{P}$, respectively. EMD is defined as the minimum cost of moving from one category to another category. The normalized earth mover distance (EMD) can be expressed as follows [47]:

(8) $$EMD\left(P,\hat{P}\right)={\left(\frac{1}{N}\left(\sum_{k=1}^{N}{\left|CDF_{p\;}\left(k\right)-CDF_{\hat{P}}\left(k\right)\right|}^{r}\right)\right)}^{1/r}$$

Here, when $\sum_{i=1}^{k}P_{di}$=$\sum_{i=1}^{k}{\hat{P}}_{di}$=1,$CDF_{p\;}\left(k\right)$ is the cumulative distribution function of $\sum_{i=1}^{k}p_{di}$.

### 4.4. Hybrid Rssi Fingerprint Model Training

In [48,49], a hybrid RSSI fingerprint training model is established in TensorFlow. Based on a ratio of 8: 2, the collected dual RF fingerprint data sets were split into a training data set and verification data set. The weight and deviation of model training were set to 0.9. The loss rate of the first fully connected layer was 0.75.

The learning rates of the convolutional layer and the last fully connected layer were set as 3 × 10^−7^ and 3 × 10^−6^, respectively. It is easier and faster to optimize the model by setting a low learning rate for the convolutional layer. Therefore, after every 10 training periods, the learning rate of all layers exponentially decays based on the decay factor of 0.95. The experiment was performed in a GPU (NVIDIA GeForce GTX 1050 Ti) (NVIDIA GeForce GTX 1050 Ti, NVIDIA, Santa Clara).

### 4.5. Calculation of Computational Complexity

The CNN structure is composed of three volume layers, three pooling layers, two fully connected layers, and one Softmax layer. In order to calculate floating point operations (FLOPs), this section deduces the formula for different layers [49]. Table 1 shows the parameters of different layers.

For a volume layer, the size of its input channel is $C_{in}{}^{i}$, the convolution kernel size is $K_{h}{}^{i}\times K_{w}{}^{i}$, the size of the output characteristic graph is $H_{i}\times W_{i}$, and the output channel is $C_{out}{}^{i}$. *i* is the ith volume layer. MAC (multiplication and addition) operand of a single-step convolution operation is as below:

(9) $$ops=C_{in}{}^{i}\cdot K_{h}{}^{i}\cdot K_{w}{}^{i}+(C_{in}{}^{i}\cdot K_{h}{}^{i}\cdot K_{w}{}^{i}-1)$$

The first item is the multiplication operand of the single convolution, and the second item is the addition operand. Considering the bias item, one item is added in Equation (9). The total number of convolution operations per convolution layer can be calculated from the dimension of the output characteristic graph, namely, $C_{out}{}^{i}\times H_{i}\times W_{i}$. The total operand is obtained from Equation (10):

(10) $$sum\_ops=(C_{in}{}^{i}\cdot K_{h}{}^{i}\cdot K_{w}{}^{i}+(C_{in}{}^{i}\cdot K_{h}{}^{i}\cdot K_{w}{}^{i}))\times C_{out}{}^{i}\times H_{i}\times W_{i}$$

The operand in the fully connected layer depends on the input feature dimension $D_{i}$ and the number of nodes in the current layer $N_{i}$. The multiplication operand is $D_{i}\times N_{i}$. The total operand in the fully connected layer is as follows:

(11) $$sum\_ops=2\cdot D_{i}\times N_{i}$$

Because there is no parameter in the pooling layer and the activation function layer, the operand is linearly related to the number of elements contained in the input tensor. The dimension of input tensor is $\left[n_{i},h_{i},w_{i},c_{i}\right]$, and the operand is expressed by Equation (12):

(12) $$sum\_ops=n_{i}\times h_{i}\times w_{i}\times c_{i}$$

The FLOPs of the model is obtained by adding the operand of each layer, and the convolution layer formula can be simplified to Equation (13):

(13) $$FLOPs_{conv}=\sum_{i=0}^{n=3}(2\cdot C_{in}{}^{i}\cdot K_{h}{}^{i}\cdot K_{w}{}^{i})\times C_{out}{}^{i}\times H_{i}\times W_{i}$$

where *n* = 3 indicates that there are three convolution layers in the model. The formula for the fully connected layer is as below:

(14) $$FLOPs_{dense}=\sum_{i=0}^{n=2}2\cdot D_{i}\times N_{i}$$

where *n* = 3 indicates that there are three fully connected layers in the model. The formula for the pooling layer and the activation function layer is as follows:

(15) $$\begin{array}{l} Flops_{pool}=\sum_{i=0}^{n=3}n_{i}\times h_{i}\times w_{i}\times c_{i}\\ Flops_{relu}=\sum_{i=0}^{n=4}n_{i}\times h_{i}\times w_{i}\times c_{i} \end{array}$$

There are three pooling layers and four relu layers. The total FLOPs are expressed in Equation (16):

(16) $$FlOPs=FLOPs_{conv}+FLOPs_{dense}+FLOPs_{pool}+FLOPs_{relu}$$

The operand of each layer and the total FLOPs are summarized in Table 2.

### 4.6. Position Estimation Algorithm

**Figure 7:**
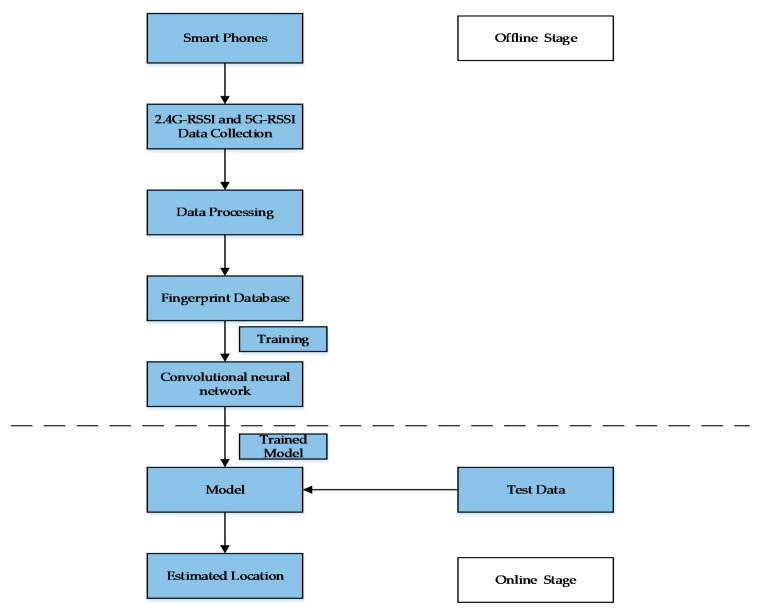
Flow chart of the location evaluation algorithm.

The location evaluation algorithm based on hybrid dual RF RSSI mainly consists of two parts: the offline part and the online part, as shown in Figure 7.

At the offline stage, multiple reference points (RP) are set, and dual RF RSSI is collected using smart phones. The collected RSSI signals are preprocessed, a dual RF RSSI fingerprint model is established, and the machine learning method is used to train the fused RSSI fingerprint. Finally, the fingerprint prediction model is generated. 

In the online stage, it is necessary to predict the physical coordinates of the TP point. The hybrid RSSI RF fingerprint collected at the TP point should be input into the hybrid RSSI fingerprint classification model to obtain the average distribution score of the point fingerprint:

(17) $$\mu =\frac{1}{N}\sum_{i=1}^{N}p_{di}\times di\;$$

Then, $\mu \times L_{max}$, the distance from the TP point to the original point, can be obtained.

**Figure 8:**
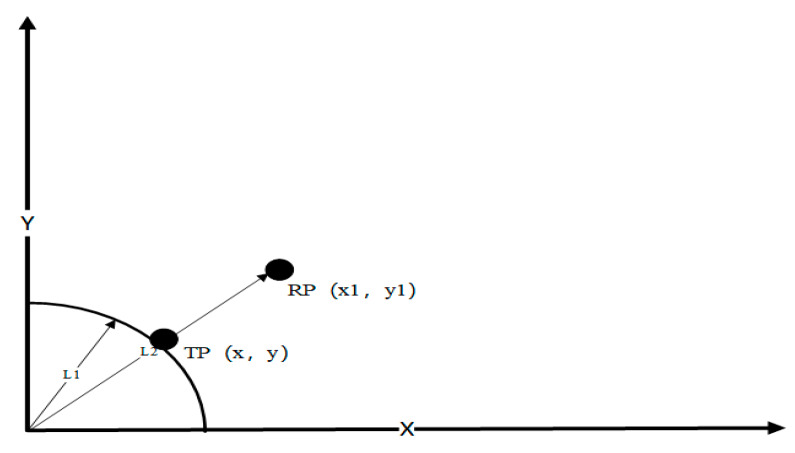
The schematic diagram of the position evaluation algorithm.

The schematic diagram of the position estimation algorithm in the online stage is displayed in Figure 8.

RP is defined as the reference node that is the closest to the TP, and its coordinates are ($x_{1},y_{1}$). The distance from the original point $L_{2}$, the coordinates of ($x_{1},y_{1}$) and $L_{2}$ are known. Moreover, TP is the prediction node, and its distance from the original point is $L_{1}$, which is also known. The coordinates (x,y) of the point should be obtained.

The equation is solved as follows:

(18) $$\frac{x}{x_{1}}=\frac{L_{1}}{L_{2}}x=x_{1}\frac{L_{1}}{L_{2}}$$

(19) $$\frac{y}{y_{1}}=\frac{L_{1}}{L_{2}}y=y_{1}\frac{L_{1}}{L_{2}}$$

In this example, the RP node is located outside the circle drawn from TP to the original point. When RP is located inside the circle, the operation principle is similar, which is not be described in detail here.

## 5. Experiment and Results

This section introduces the conditions, processes, and results of experiments. The performance of the hybrid dual radio RSSI fingerprint location method is analyzed by comparing the positioning error and the cumulative distribution function of the 2.4G-RSSI and hybrid dual radio RSSI fingerprint positioning methods.

### 5.1. Data Collection

**Figure 9:**
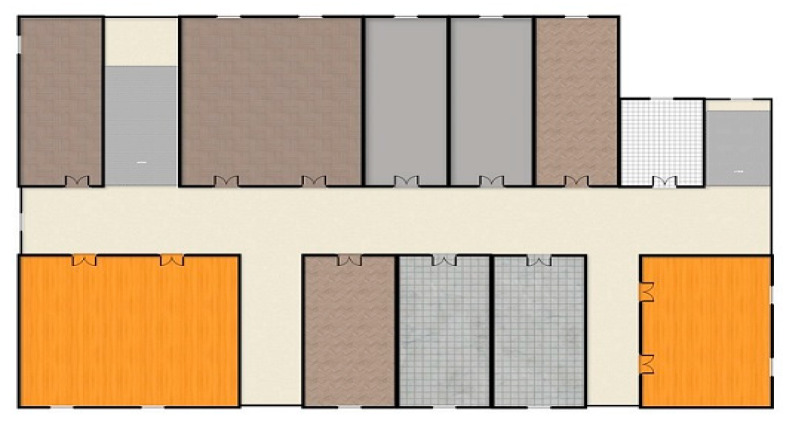
Experimental floor layout.

The experiment was conducted on the third floor of the College of Computer Science and Technology, Jilin University, Changchun, Jilin, China. The building area covers 2600 m^2^, with a total of 11 office rooms. The floor layout is shown in Figure 9. The area of the largest office is 206 m^2^, and there is a total of 7 dual radio frequency APs. The dual radio frequency RSSI reference points (RPs) are planned using a 1 × 1 m grid, and the collection area contains 11 rooms including corridors and offices.

**Figure 10:**
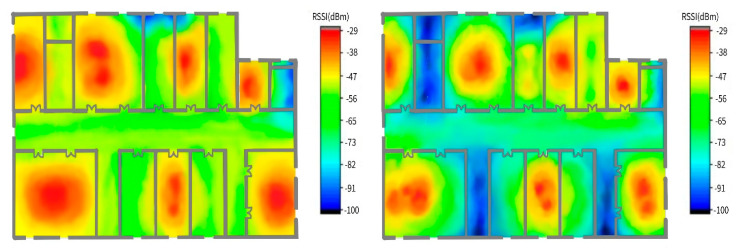
Data collection site 2.4G Wi-Fi radiation pattern and 5G Wi-Fi radiation pattern.

The experimental results demonstrate that one RP can receive the Wi-Fi RF signals of 5~7 different APs. Figure 10 shows the signal attenuation distribution (Wi-Fi AP) of the 2.4G and 5G RF RSSI. When the signal strength is >−82 dbm, the AP-2.4G RF and AP-5G RF can cover 95% and 82% of experimental scenarios, respectively. As shown in Figure 10, the RF signal distribution does not have a regular shape, indicating that it is difficult to locate targets via traditional deterministic RF attenuation localization methods.

### 5.2. Experimental Setup

During the trial phase, we performed a total of 10 position evaluation tests by randomly selecting 20 TPs each time, collecting the dual RF RSSI for each TP to form a dual RF RSSI vector, inputting these data into the CIFAR-10 position evaluation model, and finally obtaining the physical coordinates of the TPs. It is observed in the experiment that the output process of the physical coordinates of the test node can be output in real time. After 10 position evaluation tests, we average the mean, standard deviation, root mean square (RMS) value, and the maximum error of the evaluation position for each evaluation position.

### 5.3. Experimental Results and Discussion

To evaluate the performance of the HDRF location method, the HDRF RSSI fingerprint location method was compared with several traditional 2.4G Wi-Fi fingerprint indoor location algorithms using the same data set, which include the KNN [50], the support vector machines (SVM) [51], and the random forest algorithm [52]. The compared indicators include the mean, standard deviation, root mean square (RMS) value, and the maximum error of the evaluation position. The cumulative distribution functions (CDFs) of the location errors can reflect the location performances of different algorithms.

Table 3 compares the location errors of the HDRF RSSI fingerprint construction method and other traditional Wi-Fi fingerprint indoor location algorithms.

According to the above data, when the HDRF RSSI fingerprint and 2.4G-RSSI fingerprint are used, the average errors of the proposed location method are 1.7 and 2.4 m, respectively, with an improvement rate of 29.16%. When the 2.4G-RSSI fingerprint is used, the location errors of the proposed location method, the SVM (linear kernel), the KNN (k = 5), and the random forest location methods are 2.4, 2.8, 3.2, and 4 m, respectively. The errors of the proposed location method are improved by 39.3%, 42.85%, and 57.5% compared to those of the SVM, the KNN, and the random forest location methods, respectively. When the location error is 2.5, the location accuracies of the HDRF RSSI, 2.4G-RSSI, SVM (linear kernel), KNN (k = 5), and random forest are 78.2%, 62.3%, 51.7%, 23.4%, and 16.3%, respectively. When the location error is 3.5 m, the location accuracies of the algorithms are 90.6%, 73.2%, 68.3%, 69.2%, and 37.9%, respectively. The above results indicate that the HDRF RSSI fingerprint has the ability to enhance the location accuracy and location error compared with the traditional 2.4G-RSSI fingerprint method. Additionally, the proposed location method achieves a smaller average location error and higher location accuracy compared to the abovementioned algorithms. 

**Figure 11:**
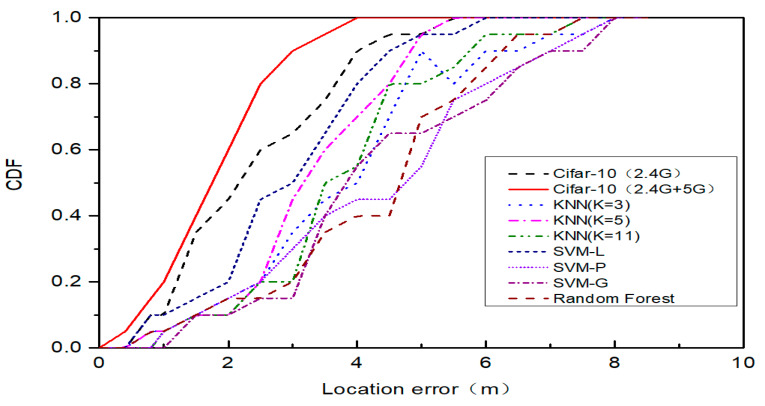
The cumulative distribution function of the location algorithm as Cifar-10, K nearest neighbor (KNN), support vector machines (SVM), and random forest.

Figure 11 shows the CDF curves of the location errors of the HDRF RSSI fingerprint construction method and several traditional Wi-Fi fingerprint indoor location algorithms.

For the KNN algorithms that are compared, in order to avoid a K value selection that couples numerical associations, we compare the K= 3 and 11 CDF curves. It can be seen that the K that is selected will have a significant impact on the results of the algorithm. When K = 11, the positioning accuracy is poorer mainly because of the large area when forecasting the training instances, which can cause too much predictor interference due to too few similar training instances, and lower the KNN positioning accuracy. When K = 3, due to the smaller K value selection, the number of equivalent examples in the training and the smaller forecast area, the forecast result is often sensitive near the reference point. If the instance point of the nearest neighbor happens to be noise, the prediction will be wrong; thus, choosing the right K value will improve the accuracy of KNN positioning.

When comparing the SVM location algorithms, the selection of the kernel function is confined by Mercer’s theorem, which hinders the further optimization of the SVM support vector regression effect and thus any further improvement of the location accuracy. When using the random forest location algorithm, the proposed fingerprint dimension of the fingerprint database is higher than the traditional 2.4G fingerprint. Overfitting may occur when using the random forest algorithm, which results in relatively low location accuracy. The positioning features are not obvious due to the relatively sparse 2.4G RF RSSI vector fingerprint features in some special areas. The distinguishability of a fingerprint near the reference point is weak, and the possibility of making the wrong estimation using the location algorithm is high, which reduces the location accuracy. Due to the existence of the Wi-Fi network communication dual radio frequency (2.4G and 5G) in the proposed HDRF RSSI fingerprint location method, the density of a fingerprint at the reference point was enhanced compared with the single radio frequency fingerprint database construction method. Meanwhile, a neural network handling method was also introduced. The hybrid radio frequency RSSI data were taken as the input of the neural network, and the distance from the node to be located to the original point was set as the output. The effect of visible and non-visual conditions on the location evaluation during the conversion of the logical distance and physical distance was solved in the positioning process of the traditional RSSI model, which contributes to the high location accuracy and precision. In the above Figure 11, the average location error and cumulative distribution function (CDF) of the proposed location algorithm is superior to those of the other algorithms.

## 6. Conclusions

As an important branch of the indoor location method, the Wi-Fi-based radio frequency RSSI fingerprint matching and location technology and probabilistic location evaluation method have been favored by Chinese and foreign scholars. In recent years, it has become the study direction and hotspot in the field of indoor positioning. This study focuses on the application of probabilistic location evaluation in Wi-Fi radio RSSI fingerprint library positioning. First, the sparsity of the traditional RP node 2.4G-RSSI fingerprint characteristics was solved. By combing 5G RF and original 2.4G radio frequency, the RP fingerprint dimension was doubled, and the distinguishability of the RP fingerprint characteristics was increased by 18.1%. Second, considering the positioning accuracy error in the conversion of logical distance and physical distance under non visual condition between TP and AP in the location evaluation stage, a hybrid dual radio frequency fingerprint model based on the machine learning method, training loss function, and location estimation algorithm were proposed. The clustering problem in the traditional location evaluation algorithm was transformed into a machine learning classification problem. Finally, a comparative experiment of different location algorithms was designed. Experimental results show that the proposed hybrid dual radio RSSI fingerprint construction method and location evaluation algorithm can effectively improve the precision and accuracy of smart phone localization in the indoor location. The accuracy of the proposed positioning method is 1.7 m. When the positioning error is 2.5 m, the positioning accuracy is 78.2%.

It should be noted that there are many factors influencing the precision and accuracy of indoor positioning based on RF communication technology, such as the complexity of spatial structure, reflection, refraction, interference, and shadow fading effect of RF signal, as well as the RF RSSI fingerprint acquisition method. Both the deterministic location method and probabilistic location method have their advantages. In future study, the universality and practicability of the proposed indoor location method will be further enhanced combined with the deterministic location method.

## Figures and Tables

**Table 1:** Parameters of different layers.

Layer	Output Dimension	Weight Parameter	Total Number of Parameters
conv2d (Conv2D)	(None, 30, 30, 32)	32 × 3 × 3 × 1 + 32	320
max_pooling2d (MaxPooling2D)	(None, 15, 15, 32)	0	0
conv2d_1 (Conv2D)	(None, 13, 13, 64)	64 × 3 × 3 × 32 + 64	18,496
average_pooling2d (AveragePooling2D)	(None, 6, 6, 64)	0	0
conv2d_2 (Conv2D)	(None, 4, 4, 64)	64 × 3 × 3 × 64 + 64	36,928
average_pooling2d_1 (AveragePooling2D)	(None, 2, 2, 64)	0	0
dense (Dense)	(None, 64)	64 × 256 + 64	16,448
dense_1 (Dense)	(None, 10)	10 × 64 + 10	650

**Table 2:** Floating point operations (FLOPs) of each layer.

Layer	FLOPs (Unit M)
conv2d (Conv2D)	0.49
relu(Relu)	0.03
max_pooling2d (MaxPooling2D)	0.03
conv2d_1 (Conv2D)	5.94
relu_1(Relu)	0.01
average_pooling2d (AveragePooling2D)	0.01
conv2d_2 (Conv2D)	1.13
relu_2(Relu)	0.001
average_pooling2d_1 (AveragePooling2D)	0.001
dense (Dense)	0.03
relu_3(Relu)	0.00006
dense_1 (Dense)	0.001
Total	7.67306

**Table 3:** Experimental test positioning error.

Classifier		Evaluation Index				
		Mean (m)	STD (m)	RMS (m)	90% (m)	MAX (m)
KNN	K = 3	3.3	1.6	4.1	5.2	7.3
	K = 5	3.2	1.2	3.6	4.8	5.0
	K = 11	4.2	1.8	4.1	5.4	7.9
SVM	Linear Kernel	2.8	1.3	3.1	4.2	5.7
	Polynomial Kernel	4.2	1.9	4.9	6.6	7.5
	Gaussian Kernel	4.3	1.9	5.1	6.9	7.6
Random Forest		4.0	2.0	4.3	6.7	7.6
Cifar-10 (2.4G)		2.4	1.3	2.7	3.7	5.4
Cifar-10 (2.4G + 5G)		1.7	0.8	2.4	2.8	3.5
